# Hydatid cyst of the hepatopancreatic groove - A case report

**DOI:** 10.1016/j.ijscr.2023.108771

**Published:** 2023-09-09

**Authors:** Zaka Ullah Jan, Nisar Ahmed, Muhammad Yunas Khan, Yusra Samin, Rija Sohail

**Affiliations:** Khyber Teaching Hospital, Peshawar, 25120, Pakistan

**Keywords:** Hydatid cyst, Hepatopancreatic groove, Case report

## Abstract

**Introduction and importance:**

Hydatid cysts are zoonoses caused by Echinococcus granulosus. It can affect any part of the body. The most common sites are the liver and lungs. Hydatid cyst of the hepatopancreatic groove is rare and to the best of our knowledge, this has not been reported in the literature before.

**Case presentation:**

We present a case of a young male patient who presented with abdomimal pain and jaundice. His workup revealed a hydatid cyst in the hepatopancreatic groove. Surgery for the hydatid cyst was done and the postoperative course was uneventful.

**Clinical discussion:**

Hepatopancreatic groove is an atypical site for a hydatid cyst. The main symptoms of a patient include vomiting, discomfort as well as pain in the epigastrium. The diagnostic imaging techniques include an ultrasound, CT scan as well as an MRI. Definitive treatment includes the cyst to be surgically removed.

**Conclusion:**

Hydatid cyst of the hepatopancreatic groove is rare. The diagnosis is usually not very easy and imaging can help in this regard. Surgery is the treatment modality of choice.

## Highlights

• Hydatid disease of the hepatopancreatic groove is rare.
• Patient presents with jaundice and abdominal pain.
• Ultrasound, CT scan and MRI helps in the diagnosis.
• Definitive treatment is pericystectomy and drainage of the cyst followed by a course of albendazole.

## 1 Introduction and importance

Hydatid cysts are zoonoses caused by adult or larval tapeworm stages of the Echinococcus granulosus genus. It is endemic in many areas of the world and can affect any part of the body, mostly liver (75 %) and lungs (15 %) [1,2].

The tapeworm stage resides in the intestine of carnivores like dogs, which makes the definitive host and the eggs are passed in the stools of infected carnivores and then eaten by herbivores like sheep. Humans make the intermediate host. Larvae develops from eggs in the intestine; and after gaining entry into the blood vessels, they can go into almost any part of our body. Most common end is the liver via the portal route, but sometimes the larvae may travel through the liver barrier and enter the lungs and all the other body organs, where they develop into small cysts [3].

The pancreas is a rare site for a hydatid disease with an incidence of 0.2 % to 2.0 % [4]. Making a definitive diagnosis in this regard may be difficult at times and thus hydatid cyst of pancreas are mostly misdiagnosed as pancreatic cystadenomas [5].

Diagnosis of hydatid cyst disease mainly depends on clinical features and epidemiological data of the disease, however ultrasound is very crucial for classifying hydatid cysts. Some types of these cysts are successfully treated with PAIR procedure (percutaneous aspiration, injection, and reaspiration) but surgery is considered as a treatment of choice [6].

The work has been reported in line with the SCARE criteria [7].

## 2 Case presentation

**Fig. 1:**
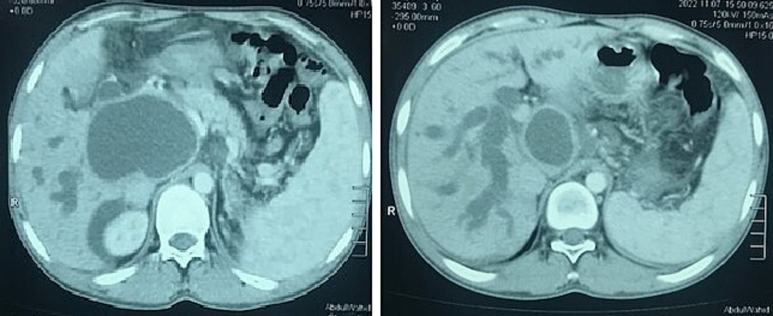
Showing the cystic lesion in the hepatopancreatic groove with intrahepatic cholestasis.

An 18-year old male patient presented to the outpatient clinic with the complaints of yellow discoloration of sclera and pain in the right hypochondrium for the last few months. The patient did not have any past medical or surgical history. He also denied any history of drugs or smoking. His family history for hydatid disease was also negative. On General Physical Examination, he was jaundiced. Upon abdominal examination, he was mildly tender in the right hyphochondrium. Rest of the systemic examination was unremarkable. His hemoglobin was 11.6 g/dl, total leukocyte count was 4.53 10 e 3/μl and Platelets count was 175 × 10 e 3/μl. His LFTs showed a total bilirubin of 16 mg/dl, SGPT of 132 U/L and ALP of 1037 U/L. His ultrasound abdomen revealed an 8.5 × 6.7 cm cystic mass in the porta hepatis near the head of the pancreas with dilatation of the intrahepatic and proximal extra hepatic biliary channels. We further worked him up. His CT scan of the abdomen showed a large thick walled 62.6 × 95.6 mm (AP X TR) cystic lesion within the hepatopancreatic groove/hepatic hilar groove with no obvious communication being evident with CBD, intrahepatic cholestasis, enlarged mesenteric nodes and ascites suspicious for duodenal duplication cyst [Fig. 1].

His CA19-9 level was normal. His serum antibodies for echinococcus weren't sent because of the radiographic imaging limitations for hydatid disease, suggesting duodenal duplication cyst in our case.

**Figs. 2 & 3:**
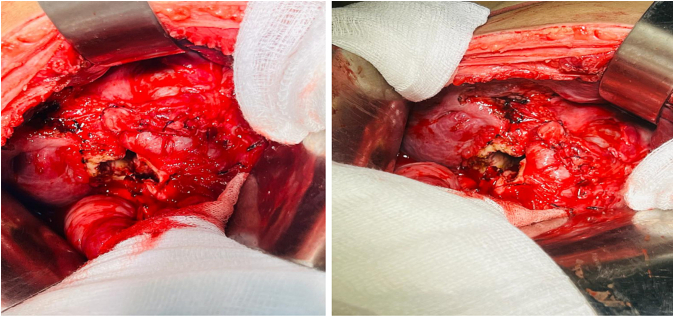

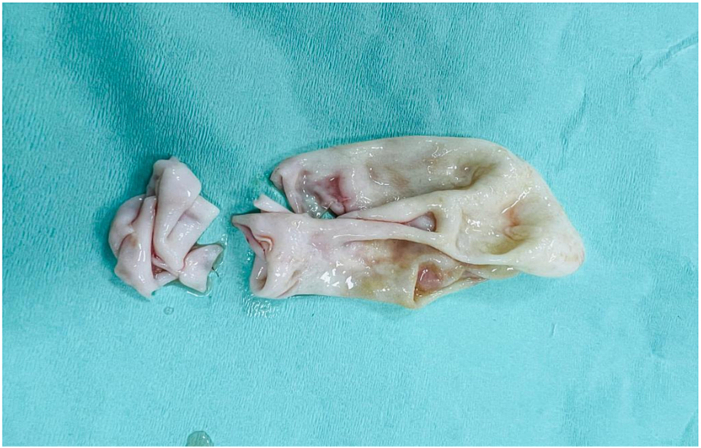
Showing hydatid cyst cavity and ruptured daughter cysts.

Following his work up, he was planned for surgery. Per-operative findings revealed hydatid cyst containing daughter cysts in the hepatopancreatic groove with dilated CBD. We were planning to remove the cyst in toto but the cyst wall was tightly adherent to the surrounding vital structures, so we had to change our operative strategy and thus pericystectomy and drainage of the cyst was done by the consultant surgeon having more than 10 years of experience after protecting the operative field and sterilization of the cyst with hypertonic saline. The residual cavity was then obliterated with omentum [Figs. 2 and 3].

Post-operatively the patient made an uneventful recovery. On 6 months follow-up, he is doing fine.

## 3 Clinical discussion

Echinococcal disease is endemic in many parts of the world. Pancreas is an atypical site for hydatid cyst to occur even in these endemic areas. When the embryo passes through the liver and lung, there is hematogenous spread of the disease. This is the most likely route of infestation of the pancreas.

Other possible but less likely routes have been suggested which include migration of embryo from the bile duct through lymphatic circulation of the intestinal mucosa into the pancreas or into pancreatic veins through portal circulation [5]. Depending on the size and location of the cyst, the clinical presentation is gradual and variable. The main symptoms include vomiting, discomfort as well as pain in epigastrium and weight loss [8]. The occurrence of obstructive jaundice is due to the cyst in the pancreatic head causing external compression of the common bile duct [9]. Rarely, abscess can form due to spontaneous rupture of the cyst into the gastrointestinal tract or the peritoneal cavity [10].

In case of suspicion of hydatid disease, diagnosis is unlikely to be made preoperatively. In some cases, diagnosis can be made by testing antibodies of echinococcus as well as imaging studies along with high clinical suspicion. The diagnostic imaging techniques include an ultrasound, CT scan as well as MRI. However, these methods have limited sensitivity due to possible overlapping of imaging features [11]. Our case also had this diagnostic dilemma and that was the reason that our case was confirmed per-operatively.

Definitive treatment includes the cyst to be surgically removed. In order to prevent dissemination, the cyst is sterilized prophylactically with a scolicidal solution and protection of the operative field is also necessary [12].

Based on the location of cyst, there are various methods of surgical treatment. For cyst located in the pancreatic head, pericystetcomy along with drainage of residual cavity is the treatment of choice [13]. An alternative to surgery in high risk surgical patients is the percutaneous drainage of cyst along with medical chemoprophylaxis with albendazole [10].

## 4 Conclusion

Hydatid cyst of the hepatopancreatic groove is rare. The diagnosis needs a high level of suspicion along with some help from the imaging studies, preferably CT scan. The treatment modality of choice is pericystectomy and drainage of the cyst followed by a course of albendazole.

## Patient perspective

I was alright when suddenly one day I had pain in the right side of my tummy. In addition to that, I had a yellow tinge to my eyes which was noticed by my family members initially.

I went to the hospital where I was thoroughly examined and certain tests were done. I was told that my ultrasound and CT scan reports show a cystic mass in my tummy which needs to be removed through an operation. Hence, after my consent, the surgical team operated upon me and took out the cystic mass. My operation was uneventful and I was sent home after staying a few nights at the hospital. I am truly grateful to the surgical team at Surgical ‘A’ unit of Khyber Teaching Hospital for taking good care of me. I am alright now and doing my normal daily life activities.

## Consent

Written informed consent was obtained from the patient for publication of this case report and accompanying images. A copy of the written consent is available for review by the Editor-in-Chief of this journal on request.

## Provenance and peer review

Not commissioned, externally peer-reviewed.

## Ethical approval

Ethical approval for this study (Ethical Committee N° 314/ADR/KMC) was provided by the Ethical committee of Khyber Teaching Hospital Peshawar on 14 December 2022.

## Funding

There are no sources of funding.

## Author contribution

Writing the paper: Dr. Zaka Ullah Jan.

Assisted in writing manuscript: Dr. Nisar Ahmed.

Literature Review: Dr. Muhammad Yunas Khan.

Designed the study: Dr. Yusra Samin.

Review of the manuscript: Dr. Rija Sohail.

## Guarantor

Zaka Ullah Jan.

## Registration of research studies

N/A.

## Declaration of competing interest

There are no conflicts of interests.
